# The efficiency of Vpx-mediated SAMHD1 antagonism does not correlate with the potency of viral control in HIV-2-infected individuals

**DOI:** 10.1186/1742-4690-10-27

**Published:** 2013-03-05

**Authors:** Hangxing Yu, Shariq M Usmani, Alexandra Borch, Julia Krämer, Christina M Stürzel, Mohammad Khalid, Xuehua Li, Daniela Krnavek, Marchina E van der Ende, Albert D Osterhaus, Rob A Gruters, Frank Kirchhoff

**Affiliations:** 1 Institute of Molecular Virology, Ulm University Medical Center, Ulm 89081, Germany; 2 Department of Internal Medicine, Erasmus Medical Center, Rotterdam, The Netherlands; 3 Department of Virology, Erasmus Medical Center, Rotterdam, The Netherlands

**Keywords:** AIDS, HIV-2, Vpx, SAMHD1, Viral immune sensing

## Abstract

**Background**

The presence of a *vpx* gene distinguishes HIV-2 from HIV-1, the main causative agent of AIDS. Vpx degrades the restriction factor SAMHD1 to boost HIV-2 infection of macrophages and dendritic cells and it has been suggested that the activation of antiviral innate immune responses after Vpx-dependent infection of myeloid cells may explain why most HIV-2-infected individuals efficiently control viral replication and become long-term survivors. However, the role of Vpx-mediated SAMHD1 antagonism in the virological and clinical outcome of HIV-2 infection remained to be investigated.

**Results**

Here, we analyzed the anti-SAMHD1 activity of *vpx* alleles derived from seven viremic and four long-term aviremic HIV-2-infected individuals. We found that effective Vpx-mediated SAMHD1 degradation and enhancement of myeloid cell infection was preserved in most HIV-2-infected individuals including all seven that failed to control the virus and developed AIDS. The only exception were *vpx* alleles from an aviremic individual that predicted a M68K change in a highly conserved nuclear localization signal which disrupted the ability of Vpx to counteract SAMHD1. We also found that HIV-2 is less effective than HIV-1 in inducing innate immune activation in dendritic cells.

**Conclusions**

Effective immune control of viral replication in HIV-2-infected individuals is not associated with increased Vpx-mediated degradation of SAMHD1.

## Background

SAM domain and HD domain-containing protein 1 (SAMHD1), the product of a gene linked to a rare and severe inherited autoimmune disease named Aicardi-Goutières syndrome [1], has recently been identified as the cellular factor that prevents HIV-1 infection in dendritic cells and macrophages [2-4]. SAMHD1 is a dGTP-stimulated triphosphohydrolase that converts deoxynucleoside triphosphates (dNTPs) to deoxynucleoside and inorganic triphosphate [5,6]. Thus, in contrast to other antiretroviral host restriction factors, such as TRIM-5alpha, ABOPEC3G proteins, and tetherin [7-9], SAMHD1 does not directly target viral components to suppress viral replication but seems to restrict HIV-1 infection of non-dividing cells by decreasing the dNTP pool concentration below the threshold required for effective reverse transcription [10,11].

A variety of primate lentiviruses has evolved effective antagonists of SAMHD1. The SIVsmm/HIV-2 lineage and SIVs infecting drills and mandrills use their Vpx protein to induce proteolytic degradation of SAMHD1 through the CUL4A/DCAF1 E3 ubiquitin ligase complex [2-4,12-14]. Furthermore, phylogenetically distinct Vpr proteins of SIVs infecting De Brazza`s, Mustached, Grivet and Vervet monkeys also evolved the capability of antagonizing SAMHD1 [13]. In contrast, HIV-1 and its direct simian counterpart (SIVcpz infecting chimpanzees) do not encode Vpx [15] and the HIV-1 Vpr protein does apparently not counteract SAMHD1-mediated restriction [3,4]. It has been reported that HIV-1 is effectively sensed by infected dendritic cells to initiate a potent antiviral immune response if the resistance of these cells to HIV-1 infection is circumvented by delivery of Vpx [16]. Viral sensing was dependent on the interaction of newly synthesized viral p24 capsid protein with cyclophylin A (CypA) and involved phosphorylation of IRF3 [16]. Thus, HIV-1 may not have evolved an antagonist of SAMHD1 to avoid infection of myeloid cells and thus to escape from immune surveillance [16].

In contrast to HIV-1-infected individuals, the majority of individuals infected with HIV-2 develop broad and effective humoral and cellular immune responses [17-20] and become long-term non-progressors with undetectable viral loads [21-24]. As a consequence, the incidence of HIV-2 infection is declining, while HIV-1 continues to expand globally [25]. HIV-2 encodes Vpx and it has been reported that this virus can efficiently infect and activate monocyte-derived dendritic cells in a CypA dependent manner [16]. These results raise the possibility that efficient virus infection of myeloid cells due to potent Vpx-mediated antagonism of SAMHD1 may activate protective immune responses and play a role in the control of viral loads in HIV-2-infected individuals [16]. To address this issue, we examined the functional activity of twenty *vpx* alleles derived from eleven HIV-2-infected individuals that differed drastically in the control of viral replication. Our analyses showed that most *vpx* alleles from both seven highly viremic and four long-term aviremic HIV-2-infected individuals efficiently degrade SAMHD1 and promote macrophage infection. The only exception were two *vpx* alleles from HIV-2 isolates derived from an aviremic patient (RH2-3) [26]. Both predicted a K68M mutation in a nuclear localization motif that disrupted the SAMHD1 degradation function. We also examined the effect of HIV-1 and HIV-2 on dendritic cell activation and found that the latter induced lower levels of CD86 expression and IFN-γ secretion. Altogether, our results suggest that efficient Vpx-mediated SAMHD1 antagonism is advantageous for viral replication and not associated with effective immune control in HIV-2-infected individuals.

## Results

### Origin and sequence analysis of HIV-2 *vpx* alleles

The 20 *vpx* alleles analyzed in the present study were derived from eleven HIV-2-infected individuals most of them living near Rotterdam and belonging to West African immigrant communities [26-31]. One infected individual (PH2) with detectable viremia was born and lived in France [31]. The other patients were born in Ghana or the Cape Verdean Islands, with the exception of RH2-26 who is Caucasian [26-30]. Some virological and immunological characteristics of these HIV-2-infected individuals have been previously described [26-31] and are summarized in Table 1. Four HIV-2-infected individuals, hereinafter referred to as effective controllers (ECs), maintained high CD4+ T cell counts (>550/μl) and undetectable viral loads (<500 viral RNA copies/ml) for 7.3 to 12.6 years, before virus isolation for biological virus cloning [26-31]. Three of these four ECs are still aviremic without treatment in 2012 (approximately ten years after isolation of the viruses used in this study). In contrast, the seven viremic HIV-2-infected individuals, named non-controllers (NCs), generally had low CD4 counts (<240/μl) and most of them suffered from end stage AIDS at the time of virus isolation (Table 1). RNA loads were available for five of the seven individuals with progressive HIV-2 infection and ranged from 4.36 to 5.52 log_10_ copies/ml. Thus, the EC and NC groups of HIV-2-infected individuals differed drastically in their ability to control viral replication.

**Table 1:** Overview on HIV-2 samples analyzed

**Subject**	**Clones**	**CD4**^**a**^	**PVL**^**b**^	**Group**^**c**^	**R5/X4**^**d**^	**Group**	***vpx *alleles**^**e**^	**References**
RH2-3	2C5/8A3	770	bql	aviremic	R5	A	3/3	[26-30]
RH2-13	1D4/5C1	900	bql	aviremic	R5	A	3/3	[26-30]
RH2-14	1B1/1D1	550	bql	aviremic	R5	A	3/3	[26-30]
RH2-22	2C2/1B4	670	bql	aviremic	R5	B	4/4	[26-30]
RH2-1	A8/D8	240	>500	viremic	R5	A	3/6	[26-30]
RH2-5	2D11/2F10	120	110,000	viremic	R5	A	3/3	[26-30]
RH2-7	B3/D6	10	>500	viremic	R5	A	3/3	[26-30]
RH2-21	2B2/2F9	60	59,000	viremic	n.k.	A	3/3	[26-30]
RH2-24	2F11	70	330,000	viremic	R5/X4	A	3	[26-30]
RH2-26	1C1	10	>500	viremic	R5/X4	A	8	[26-30]
PH2.1	C12/E6	200	>500	viremic	n.k.	A	3/3	[30,31]

^a^ Absolute CD4 T cell counts per μl blood.

^b^ Copies of plasma viral RNA per milliliter.

^c^ Grouped based on VLs.

^d^ Usage of CCR5 or CXCR4 for viral entry.

^e^ Number of *vpx* alleles sequenced per biological HIV-2 clone.

Abbreviations: *PVL* plasma viral load, *Bql* below quantifiable limit, *n.k*. not known.

**Figure 1:**
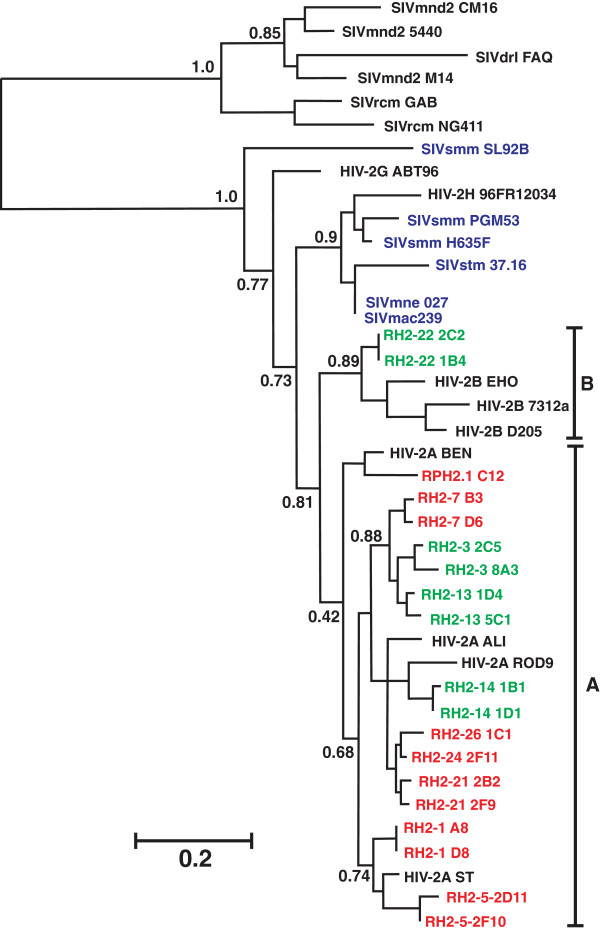
**Evolutionary relationships among HIV-2 and SIV Vpx amino acid sequences.** HIV-2 Vpx sequences newly analyzed in the present study are highlighted in green (ECs) and red (NCs). Numbers on branches are percentage estimated posterior probabilities.

To examine whether differences in virus control in HIV-2-infected individuals are associated with differences in Vpx function, we amplified PCR fragments encompassing the *vpx* genes from biological HIV-2 clones obtained from the patient samples. As described previously [26-30], these HIV-2 clones were generated by co-cultivation of limiting dilutions of PBMCs from HIV-2-infected donors with PBMCs from seronegative donors. A total of 70 *vpx* genes (3 to 8 for each HIV-2 clone) were sequenced. As expected, *vpx* alleles derived from the same biological clone of HIV-1 were highly homologous or identical. A total of 20 alleles that encoded the respective consensus Vpx amino acid sequence of each of the twenty biological HIV-2 clones were inserted into a CMV-promoter-based vector [32], which co-expresses Vpx and eGFP from a bi-cistronic RNA. To ensure that the *vpx* alleles were representative for each patient, we analyzed two biological HIV-2 clones from most individuals, except for RH2-24 and RH2-26, where only single biological clones were available for analysis (Table 1). Sequence and phylogenetic analyses verified that all expression constructs contained the desired HIV-2 *vpx* alleles and showed that those from ECs and NCs did not form distinct clusters (Figure 1). All HIV-2 strains analyzed belonged to group A, with the exception of RH2-22 that clustered with HIV-2 group B strains (Figure 1).

**Figure 2:**
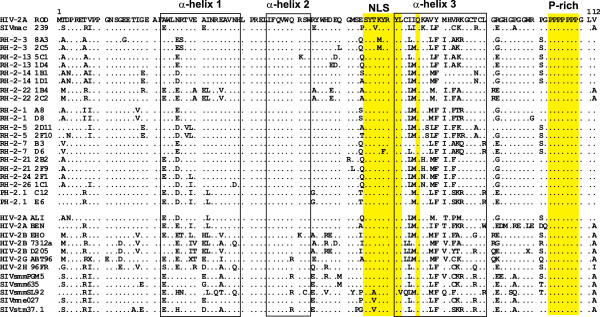
**Alignment of HIV-2 and SIV Vpx sequences.** The HIV-2 ROD Vpx sequence is shown on top for comparison. The three putative α-helical regions, the nuclear localization signal (NLS), residue Q76 involved in DCAF1 binding, and the C-terminal proline-rich region are indicated. Dots indicate amino acid identity.

Alignment of the deduced amino acid sequences showed that all *vpx* genes analyzed contained intact reading frames that predicted full-length Vpx protein sequences (Figure 2). The HIV-2 Vpx sequences showed a high degree of conservation and those derived from the same patient frequently only differed by single amino acid changes. Several domains and residues previously described to be critical for Vpx function, such as the C-terminal stretch of seven proline residues that are critical for stable Vpx expression [33,34], as well as a Wx4Φx2Φx3AΦxH motif and residue Q76 involved in DCAF1 binding [35-37] were preserved in all HIV-2 Vpx alleles analyzed, as well as in published group A, B, G and H HIV-2 and SIVsmm/mac/mne Vpx amino acid sequences (Figure 2). Unexpectedly, however, some variations were detected in a nuclear localization signal (NLS) (SYTKYRYL) signal, which is important for nuclear import of Vpx [38-42]. Most notably, all *vpx* alleles obtained from patient RH2-3 predicted a SYTKYRYL to SYTMYRYL change (K68M) in this NLS (Figure 2). Furthermore, *vpx* alleles derived from the RH2-7 D6 biological HIV-2 clone predicted an adjacent Y69F substitution.

**Figure 3:**
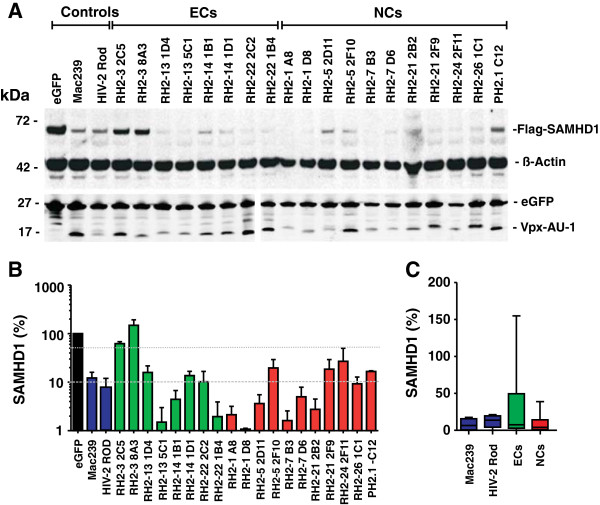
**Most HIV-2 Vpx proteins from controllers and non-controllers of virus replication degrade SAMHD1.** (**A**) SAMHD1, ß-actin, eGFP control and Vpx-AU-1 levels in HeLa cells stable expressing Flag-SAMHD1 with pCGCG constructs expressing the indicated HIV-2 Vpx proteins, the control HIV-2 ROD or SIVmac239 Vpx alleles or only eGFP. Protein expression levels were determined by western blot two days post-transfection. (**B**) Average levels of SAMHD1 in the presence of the indicated HIV-2 Vpx proteins. The SAMHD1 signals were obtained by western blot analysis as described in panel A, quantified by Licor Odyssey software and normalized to the ß-actin signals. The graph shows mean values and standard deviation (SD) of normalized SAMHD1 expression levels from four independent experiments. HIV-2 *vpx* genes were grouped based on the viral loads of the patients and are color coded green (ECs) or red (NCs). (**C**) The levels of SAMHD1 expression were determined in stable transfected HeLa cells in the presence of *vpx* alleles from HIV-2-infected ECs and NCs and are shown relative to those measured in the absence of Vpx (100%).

To determine the potency of *vpx* alleles from HIV-2-infected individuals in degrading SAMHD1, we transfected HeLa cells stably expressing Flag-tagged SAMHD1 with pCGCG constructs coexpressing Vpx and eGFP and analyzed them by Western blot. Since no broadly reactive HIV-2 Vpx-specific antibody is available, all Vpx proteins were expressed with an C-terminal AU-1 tag in order to monitor protein expression levels. In agreement with published data [2-4], expression of the control SIVmac239 and HIV-2 ROD Vpx proteins strongly reduced the steady-state expression levels of SAMHD1 compared to cells transfected with the pCGCG construct expressing only eGFP (Figure 3A). All Vpx proteins were expressed at detectable levels and the great majority of them (18 of 20) clearly reduced the levels of SAMHD1 expression, albeit with variable efficiency. Only the RH2-3 2C5 and 8A3 *vpx* alleles that predicted the K68M mutation in the NLS were inactive in degrading SAMHD1 (Figure 3A). In contrast, the RH2-7 D6 Vpx that contains the adjacent Y69F change efficiently degraded SAMHD1 (Figure 3A).

Quantitative analyses showed that HIV-2 Vpx proteins reduced the levels of SAMHD1 expression by up to two orders of magnitude (Figure 3B). Some patient-derived HIV-2 Vpx alleles, such as RH2-13 5C1 and RH2-1 D8, were on average even more potent in mediating SAMHD1 degradation than the control HIV-2 ROD and SIVmac239 Vpx proteins. In contrast, the two HIV-2 RH2-3 2C5 and 8A3 Vpx alleles containing the K68M change were inactive against SAMHD1 (Figure 3B). On average, *vpx* alleles derived from NCs were more active in degrading SAMHD1 compared to those obtained from ECs (8.4±1.6%, n=39 vs 31.7±9.2%, n=25; values gives percentages of SAMHD1 expression levels compared to the eGFP control [100%] and represent means±SEM) (Figure 3C). However, this difference was just due to the lack of activity of the *vpx* alleles derived from the EC RH2-3 (105.4±14.9) and failed to reach significance. Thus, Vpx-mediated SAMHD1 degradation was usually preserved in HIV-2-infected indIviduals irrespectively of the efficiency of virus control.

### Vpx alleles from viremic and aviremic individuals promote macrophage infection

**Figure 4:**
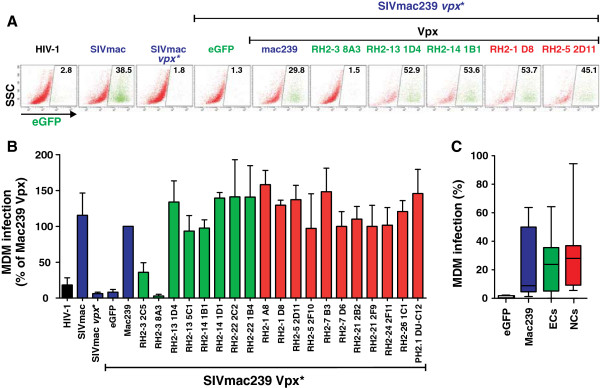
**HIV-2 Vpx-mediated enhancement of virus infection of macrophages.** (**A**) Macrophages were transduced with the VSV-G pseudotyped wild-type HIV-1 NL4-3 and SIVmac239 IRES-eGFP virions (panels 1 and 2) or a VSV-G pseudotyped *vpx*-defective SIVmac239 IRES-eGFP construct produced in the presence of pCGCG vectors expressing eGFP alone (panel 3) or together with the indicated Vpx proteins (panels 4–10). Virus stocks were produced by transient transfection of 293T cells. High infection rates were associated with increased rates of apoptosis and thus reduced numbers of cells analyzed in some experiments. (**B**) Average percentages and SDs of virally infected GFP+ cell levels detected in macrophages derived from four different donors quantified by flow cytometric analysis at four days post-transduction. Virus infectivity was normalized to infection of TZM-bl indicator cells. (**C**) Enhancement of macrophages infection by *vpx* alleles derived from HIV-2-infected individuals grouped based on their viral loads and the infecting viruses. See legend to figure 3 for further detail.

The results described above suggested that most HIV-2 Vpx proteins are active in promoting infection of myeloid cells by degrading SAMHD1. To directly determine this, we first generated a *vpx* defective IRES-eGFP reporter construct of SIVmac239. Similarly to previously described HIV-1 NL4-3-based IRES-eGFP constructs [43,44], this SIVmac239-based construct co-expresses Nef and eGFP via an internal ribosome entry site and thus allows the convenient identification and quantification of virally infected cells by flow cytometric analysis (examples shown in Figure 4A). The SIVmac239 molecular clone was selected for these studies because it is well characterized and expresses a functional Vpx protein [3,4] that is highly homologous to those of HIV-2 strains. Furthermore, SIVmac and HIV-2 belong to the same lineage of primate lentiviruses and both are genetically closely related and originated from SIVsmm infecting sooty mangabeys [45,46]. Virus stocks were generated by cotransfection of 293T cells with a *vpx-*defective SIVmac239 IRES-eGFP construct and pCGCG-based vectors expressing the various HIV-2 Vpx proteins or eGFP alone as a negative control. Predictably, mutation of *vpx* severely impaired and co-expression of the mac239 Vpx in the virus producer cells restored the capability of SIVmac239 to infect primary human monocyte-derived macrophages (MDM) (Figure 4A, B). The great majority of HIV-2 Vpx proteins from both NCs and ECs efficiently rescued macrophage infection (Figure 4B). Predictable exceptions were the 8A3 and 2C5 *vpx* alleles from individual RH2-3 that were unable to degrade SAMHD1 in HeLa cells (Figure 3). Thus, all HIV-2 Vpx proteins that degraded SAMHD1 in transfection assays were also capable of promoting macrophage infection. No antibodies against these patient-derived Vpx proteins are available and the signals obtained with the antibody against the AU-1 tag were too weak to quantify virion incorporation (data not shown). However, our finding that coexpression of a great majority of these HIV-2 Vpx proteins *in trans* potently increased the ability of the *vpx*-defective SIVmac construct to infect macrophages implies that they were incorporated in the viral particles. On average, *vpx* alleles from ECs and NCs of HIV-2 infection were equally active in promoting macrophage infection (30.9±3.6%, n=24 vs 30.3±3.7, n=45; values give mean ± SEM) (Figure 4C).

### Substitution of K68M in the NLS impairs HIV-2 Vpx function

**Figure 5:**
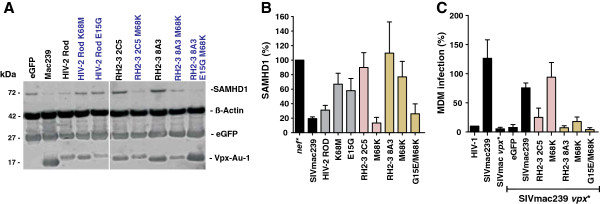
**Effect of naturally occurring mutations in the NLS of HIV-2 Vpx proteins on SAMHD1 degradation and macrophage infection.** (**A**) HeLa cells stably expressing FLAG tagged SAMHD1 were mock transfected or transfected with constructs expressing the indicated AU-1 tagged Vpx proteins for 48 h before whole-cell extraction and analysis by western blot using the indicated antibodies. (**B**) Quantitation of Vpx-mediated degradation of SAMHD1. Shown are the average levels and SEM of SAMHD1 expression in Hela cells in the presence of the indicated Vpx alleles. Results were derived from four independent experiments. (**C**) MDM isolated from four donors were transduced by VSV-G-pseudotyped SIVmac239 eGFP reporter viruses produced in the presence of the indicated Vpx proteins and GFP^+^ MDM were quantified four days later.

As described above, two *vpx* alleles (RH2-3 8A3 and 2C5) had little if any effect on SAMHD1 expression levels and failed to promote macrophage infection. To determine whether the K68M substitution was responsible for their lack of activity, we mutated it back to 68K found in the consensus NLS sequence (Figure 1). As shown in Figure 5A, all AU-1-tagged Vpx proteins were expressed at detectable levels. Substitution of M68K reduced the expression levels of the RH2-3 2C5 Vpx but fully restored its capability to degrade SAMHD1 and to promote macrophage infection (Figure 5B, 5C). In comparison, substitution of M68K in the RH2-3 8A3 Vpx increased these activities only marginally. The 8A3Vpx differs from 2C5 by a total of four amino acid changes (Figure 1). However, only a single substitution of E15G distinguishes the 8A3 M68K Vpx from functionally active Vpx proteins. Indeed, combined substitutions of G15E and M68K restored the ability of the RH2-3 8A3 Vpx to degrade SAMHD1 (Figure 5A, 5B). In further support of a functional role of E15, substitution of E15G impaired the anti-SAMHD1 activity of the HIV-2 ROD Vpx function. Unexpectedly, however, RH2-3 8A3 Vpx containing the G15E and M68K changes remained inactive in promoting MDM infection (Figure 5C) suggesting that it may not be efficiently incorporated into progeny virions.

To determine the effect of alterations in the NLS of HIV-2 Vpx proteins on the cellular distribution of Vpx and SAMHD1, we transfected different Vpx expression constructs into HeLa cells which were stably expressing FLAG tagged SAMHD1 and analyzed them by laser scanning confocal microscopy. In agreement with published data [4,47], SAMHD1 localized mainly to the nucleus in the absence of Vpx (Additional file 1: Figure S1, Additional file 2: Figure S2). In cells expressing the SIVmac239 Vpx, the residual traces of SAMHD1 co-localized with Vpx within yet-to-be-defined cytoplasmic compartments (Additional file 1: Figure S1, Additional file 2: Figure S2 and data not shown). Unexpectedly, the HIV-2 RH2-3 2C5 Vpx, which contains the K68M substitution, re-localized SAMHD1 from the nucleus to the cytoplasm but was largely unable to degrade it (Additional file 2: Figure S2D). In contrast, the second Vpx allele derived from individual RH2-3 (8A3) localized throughout the cell and lacked both the ability to re-localize and to degrade SAMHD1 (Additional file 2: Figure S2F). Substitution of M68K resulted in a strongly localized distribution of the RH2-3 2C5 Vpx protein at the cell’s edge and restored its capability to degrade SAMHD1 (Additional file 2: Figure S2E). In comparison, the RH2-3 8A3 M68K Vpx that displayed only modest anti-SAMHD1 activity (Figures 3 and 4) showed diverse effects; in some cells residual SAMHD1 was detected in the nucleus (Additional file 1: Figure S1, Additional file 2: Figure S2G and data not shown). Finally, combined substitutions of G15E and M68K resulted in cytoplasmic localization of Vpx and effective degradation of SAMHD1 (Additional file 1: Figure S1, Additional file 2: Figure S2H).

The results obtained with the two RH2-3 HIV-2 *vpx* alleles suggested that the effect of the K68M change is to some extent context dependent. To further examine this, we also analyzed an HIV-2 ROD Vpx mutant containing this substitution. The parental ROD Vpx showed a punctuated cytoplasmic distribution and reduced the levels of SAMHD1 expression below the detection limit (Additional file 3: Figure S3A). Mutation of K68M resulted in distribution of the ROD Vpx throughout the cell and impaired its ability to degrade SAMHD1 (Additional file 3: Figure S3B). In comparison, substitution of E15G did not affect the cytoplasmic localization of the ROD Vpx but impaired its effect on SAMHD1 (Additional file 3: Figure S3C). Altogether, these results show that residue K68 plays a key role in Vpx-mediated SAMHD1 degradation and the subcellular localization of Vpx.

**Figure 6:**
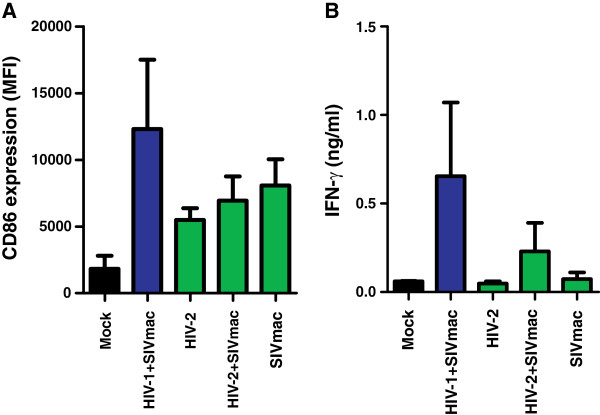
**Effect of HIV-1 and HIV-2 on DC activation.** (**A**) CD86 surface expression in DCs after infection with HIV-1 and HIV-2 IRES-eGFP constructs alone or in combination with VSV-G-pseudotyped SIVmac239 particles. The levels of CD86 expression by virally infected (eGFP+) cells were measured at 3 days post-infection. Panels **A** and **B** show mean values (±SEM) derived from four experiments. HIV-1 alone did not infect DCs at detectable levels. (**B**) Levels of IFN-γ in the supernatant of the infected DC cultures.

It has been previously suggested that HIV-2 may be effectively controlled in most infected individuals because it activates innate antiviral immunity in dendritic cells [16]. To examine whether HIV-2 can activate DCs, we transduced these cells with VSV-G-pseudotyped HIV-1 NL4-3 and HIV-2 ROD IRES-eGFP constructs in the presence or absence of SIVmac239. As expected from previous data [16], HIV-1 alone was unable to infect DCs and “enforced” infection resulted in high levels of CD86 expression by HIV-1-infected DCs (Figure 6A) and the secretion of high levels of IFN-γ in the culture supernatant (Figure 6B). In comparison, HIV-2-infected cells showed substantially lower levels of CD86 expression than those infected with HIV-1 (Figure 6A). Similarly, only low levels of IFN-γ were detected in DC cultures infected with HIV-2 and/or SIVmac (Figure 6B) although the infection rates were similar in HIV-1/SIVmac (7.6±1.7%, n=4; mean value ± SEM) and HIV-2/SIVmac (9.4±2.1%, n=4) exposed cultures. Thus, similarly to SIVmac, HIV-2 did not induce efficient activation and antiviral type I interferon responses in infected DC cultures.

## Discussion

In the present study, we examined the function of Vpx proteins derived from seven viremic HIV-2-infected AIDS patients (NCs) and four long-term aviremic individuals (ECs) who did not develop immunodeficiency. We found that efficient Vpx-mediated degradation of SAMHD1 to promote myeloid cell infection is usually preserved in both HIV-2-infected individuals that efficiently control the virus as well as in individuals that develop high viral loads and disease. SAMHD1 degradation could have both positive or negative effects on the efficiency of HIV-2 replication *in vivo*. On the one hand it allows efficient infection of macrophages and dendritic cells and thus expands the number of viral target cells. On the other hand it has been suggested that the efficiency of SAMHD1 degradation by Vpx may contribute to the effective control of viral replication in HIV-2 infected individuals because viral immune sensing by infected dendritic cells may induce potent immune responses [16]. In fact, particularly broad and potent innate and adaptive immune responses have been observed in HIV-2-infected individuals [17-20] and most of them efficiently control viral replication and become long-term non-progressors [21-25]. However, our finding that major differences in the efficiency of control of HIV-2 replication in infected individuals were not associated with significant differences in the potency of Vpx-mediated SAMHD1 degradation argues against a major role of this mechanism in the virological and clinical outcome of HIV-2 infection. Notably, it has recently been shown that polymorphisms in SAMHD1 are not associated with immune control in HIV-1-infected individuals, although it remained to be determined whether these polymorphisms affect viral infection of myeloid cells and immune sensing *in vitro*[48].

It has been previously reported that HIV-2 infection activates dendritic cells (DCs) in a CypA dependent manner and thus suggested that efficient infection and activation of DCs may contribute to the effective control of viral replication in most HIV-2 infected individuals [16]. If this hypothesis is correct, HIV-2 strains that are associated with high viral load and AIDS (similarly to most HIV-1 infections) should show a reduced capability to infect myeloid cells and thus lack a potent SAMHD1 antagonist (like HIV-1). We found, however, that this is not the case because Vpx-mediated degradation of SAMHD1 was preserved in all biological viral clones derived from seven HIV-2-infected individuals that developed high viral loads and progressed to AIDS. These data suggest that Vpx-mediated degradation of SAMHD1 to facilitate myeloid cell or resting T cell infection is usually advantageous for HIV-2 replication *in vivo* and not associated with improved immune control. In agreement with this possibility, we found that (unlike HIV-1) HIV-2 does not efficiently activate antiviral innate immunity in infected dendritic cells. It has been shown that HIV-1-induced activation of innate anti-viral immunity in DCs requires the interaction of newly synthesized viral capsid with CypA [16]. Whether or not the HIV-2 capsid can bind to CypA is under debate. One study reported interaction, albeit with much lower affinity than the HIV-1 capsid [49]. Others, however, documented that neither HIV-2 nor SIVmac recruit CypA into their cores and showed that drugs that block CA-CypA interactions do not affect the titers of these viruses [50]. In agreement to the results of the previous report [16], we found that VSV-G-pseudotyped SIVmac239 virus-like particles alone did not efficiently induce dendritic cell activation (Figure 6). SIVmac and HIV-2 are closely related and have a common ancestor with SIVsmm infecting sooty mangabeys [45,46,51]. Thus, it seems plausible that both do not activate antiviral innate immunity in human dendritic cells, particularly since one would expect selection against effective immune sensing during adaptation of HIV-2 to humans. More comprehensive comparative studies on innate immune sensing of various HIV-1 and HIV-2 strains will be necessary to clarify this issue. Notably, it has been reported that DCs are actually more susceptible to HIV-1 than to HIV-2 infection [52]. Thus, further studies using primary HIV-2 strains seem highly warranted, particularly since delivery of large quantities of Vpx by viral-like particles (VLPs) into the viral target cells bears the risk of artifacts. In either case our findings that the potency of SAMHD1 antagonism and virus control do not correlate and that HIV-2 did not efficiently activate DCs, suggest that immune sensing of HIV-2 by infected human DCs may be usually too ineffective to induce protective immune responses.

The only two *vpx* alleles that were inactive in antagonizing SAMHD1 and failed to boost myeloid cell infection were both derived from individual RH2-3, who efficiently controlled viral replication. This preliminary finding suggests that counteraction of SAMHD1 may be counter-selected against in a small subset of HIV-2-infected individuals. Thus, it will be interesting to further examine whether, similarly to HIV-1 [16], a minority of HIV-2 strains may activate antiviral innate immunity in DCs and thus lose the SAMHD1 degradation function of Vpx to facilitate viral immune evasion. The viral accessory proteins are well known for their functional plasticity [7-9] and some primate lentiviruses utilize Vpr to antagonize SAMHD1 [13]. Thus, the possibility that some HIV-2 strain may utilize Vpr instead of Vpx to counteract SAMHD1 can also not be dismissed entirely and may warrant further investigation. It will also be of interest to determine whether Vpx-mediated counteraction of SAMHD1 may be counter-selected against in individuals with dual HIV infection. Co-infection of DCs with SIVmac and HIV-1 *in vitro* resulted in efficient activation and induction of a type I IFN response because delivery of Vpx by SIV virions eliminates the restriction to HIV-1 infection [16]. It is conceivable that HIV-2-mediated delivery of Vpx into DCs may also increase their susceptibility to HIV-1 infection *in vivo* in dually infected individuals. The resulting increased activation of innate and adaptive immune responses may enhance viral control and promote the selection of HIV-2 variants lacking the SAMHD1 degradation function of Vpx. Recently, it has been reported that an HIV-1 superinfection in an HIV-2-infected women resulted in efficient control of HIV-1 replication [53]. This case is quite remarkable because the patient did not show HIV-associated disease and maintained low HIV-1 and undetectable HIV-2 RNA levels during the entire observation period of six years and in the absence of anti-retroviral therapy [53]. It would have important implications for vaccine development and strategies to improve immune control if increased HIV-1 infection of myeloid cells by antagonism of the SAMHD1 would indeed lead to improved innate and adaptive immune responses that are associated with effective virus control. Dual HIV infections are not uncommon in West Africa [25,54,55] and future studies of Vpx function and the virological and clinical outcome of infection in these individuals seem highly warranted.

Currently, the exact mechanism of Vpx-mediated SAMHD1 antagonism is not fully understood reviewed in [56-59]. In agreement with the recent finding that Vpx-mediated degradation of SAMHD1 seems to be initiated in the nucleus [47], our data suggest that all active Vpx alleles analyzed in the present study may be capable of interacting with SAMHD1 in the nucleus to relocalize it to the cytosol of the cell for degradation. Some active Vpx proteins colocalized with SAMHD1 throughout the cytoplasm, whereas others resulted in a compartmentalized localization of Vpx and SAMHD1 towards the leading edges of the cell (Additional file 1: Figure S1, Additional file 2: Figure S2G and data not shown). An obvious limitation of these microscopic analyses is that effective SAMHD1 degradation by some Vpx proteins makes it difficult to define the subcellular localization of the former in the presence of Vpx. Thus, e.g. the possibility that SAMHD1 is efficiently degraded by Vpx in the nucleus and thus mainly detectable in the cytoplasm cannot be dismissed entirely but seems unlikely since all active Vpx protein showed a cytoplasmic localization. All six *vpx* alleles derived from two biological HIV-2 clones obtained from individual RH2-3 predicted a substitution of K68M in a putative NLS. This alteration generally disrupted the effect of Vpx on SAMHD1 and macrophage infection without reducing the steady state expression levels. However, the effects were to some extent context-dependent. The presence of K68M in the RH2-3 8A3 and ROD Vpx proteins impaired their capability to relocalize and to degrade SAMHD1 (Figure 5, Additional file 1: S1 to Additional file 3: Figure S3). In contrast, the RH2-3 2C5 Vpx relocalized SAMHD1 from the nucleus to the cytoplasm but failed to degrade it efficiently. The mechanisms underlying these different phenotypes need further investigation.

In the present study we utilized an SIVmac construct to study the effect of HIV-2 Vpx on viral infectivity for macrophages. Since this may raise the concern that some HIV-2 Vpx proteins may not be efficiently incorporated into heterologous SIVmac particles, we also attempted to generate analogous proviral constructs of the group A HIV-2 ROD clone. The wild-type HIV-2 ROD IRES-eGFP construct was infectious for macrophages and utilized in the studies on viral immune sensing (Figure 6). Unfortunately, the *vpx*-defective HIV-2 ROD derivative was generally poorly infectious even in highly susceptible TZM-bl indicator cells and thus not suitable for functional studies (data not shown). We do not feel, however, that utilization of SIVmac compromises the main conclusions of our study. It is noteworthy, that some HIV-2 strains are actually more closely related to SIVmac (that has the same origin) than to one another since HIV-2 resulted from several zoonotic transmission of SIVsmm to humans [51]. Moreover, the dileucine-containing motif (D-X-A-X-X-L-L) in the N-terminal half of p6^*gag*^ that is required for Vpx incorporation into viral particles is highly conserved in the HIV-2/SIVsmm/SIVmac group [60]. In fact, we found that 18 of 20 HIV-2 Vpx proteins efficiently increased the ability of SIVmac to infect macrophages, which implies that they were incorporated into the virions. The two inactive Vpx proteins (RH2-3 2C5 and 8A3) contained mutations in their NLS and were unable to degrade SAMHD1. In one case ineffective virion incorporation may have contributed to the lack of activity of the RH2-3 8A3 Vpx since changes of E15G and K68M restored its ability to degrade SAMHD1 but not to promote macrophage infection (Figure 5).

Our finding that all 12 *vpx* alleles derived from seven NCs degraded SAMHD1 and promoted macrophage infection strongly suggests that potent infection of myeloid cells is usually not associated with effective immune control of HIV-2. However, studies of *vpx* alleles from larger numbers of HIV-2-infected individuals are necessary to clarify whether exceptions may exist. For example, it will be interesting to determine whether HIV-2 capsids may differ in their interaction with CypA and the induction of innate immune responses and whether such differences affect the functionality of Vpx in infected individuals. Furthermore, it will be of interest to examine if co-infection with HIV-2 relieves the restriction of DCs to HIV-1 infection by Vpx-delivery and induces potent antiretroviral immune responses *in vivo.* A better understanding of how both viruses affect one another and especially whether HIV-2-assisted increased HIV-1 infection of DCs may induce protective immune responses may yield important information for immunotherapy and vaccine approaches.

## Conclusions

Our data suggest that Vpx-mediated degradation of SAMHD1 to promote myeloid cell infection is usually preserved and thus advantageous in both viremic and aviremic HIV-2-infected individuals. In agreement with this observation, we found that HIV-2 ROD does not induce potent activation of infected DCs. Together, these results suggest that differences in the efficiency of myeloid cell infection and immune sensing are not the main reason why some HIV-2-infected individuals efficiently control the virus, whereas others develop high viral loads and progress to AIDS.

## Methods

### Patients

Vpx genes were derived from eleven individuals from the Rotterdam cohort. The characteristics of these patients and the determination of CD4+ T cell numbers and plasma viral loads have been described previously [26-31] and are summarized in Table 1. Viremic HIV-2-infected individuals (NCs) were defined as having a plasma viral load >500 copies per ml, and non-viremic subjects (ECs) were defined as having viral loads <500 copies per ml. All HIV-2 infected individuals examined were seronegative for HIV-1. With the exception of one individual (RH2-22), who was infected with HIV-2 subtype B all individuals were infected with subtype A. All study participants provided informed consent and the studies were approved by the local Ethical Committees.

### Generation of HIV-2 *vpx* alleles

Genomic viral RNA genomes were extracted from biological HIV-2 clones isolated as described previously [30,31]. In brief, these HIV-2 clones were generated by co-cultivation of limiting dilutions of PBMCs from HIV-2-infected donors with PBMCs from seronegative donors. *Vpx-vpr* fragments were amplified by RT-PCR using the cDNA primers (p5^’^;vpx2, 5^`^-CAGGTACCRTCACTTCAATTYCTRGCCYTAG-3 and p3^’^vpr2, 5^`^-CTGGCAATGGTAG-CAGCATYGCTTACAATAGCA-3^`^). PCR amplification products were cloned in the TOPO-TA vector (Invitrogen) and three to eight clones were sequenced to identify one representing the consensus sequence of the respective biological HIV-2 clone. Subsequently, the selected *vpx* genes were amplified by PCR using primers introducing flanking XbaI and MluI sites and cloned into the pCGCG vector [32]. To examine the expression levels of Vpx a 3^`^primer introducing a C-terminal AU-1 tag was used for PCR amplification. The accuracy of all PCR-derived inserts was confirmed by sequence analysis.

### Proviral constructs

Generation of SIVmac239-based proviral constructs carrying a functional or disrupted *vpx* gene and a *nef* reading frame followed by an internal ribosome entry site (IRES) and the eGFP gene were generated essentially as previously described for analogous HIV-1-based constructs [43,44].

### Cell culture

293T cells, TZM-bl cells and HeLa cells stably expressing Flag-SAMHD1 were maintained in Dulbecco’s modified Eagle medium (DMEM) supplemented with 10% FCS plus 2 mM glutamine and 100 Unit/ml Penicillin-Streptomysin. PBMC from healthy human donors were isolated using lymphocyte separation medium (Biocoll Separating Solution, Biochrom), stimulated for 3 days with PHA (2 μg/ml) and cultured in RPMI1640 medium with 10% FCS and 10 ng/ml IL-2 prior to infection. MDMs were generated from PBMCs by 7 days stimulation with 50 ng/ml of either recombinant human granulocyte-macrophage colony-stimulating factor (rhGM-CSF, R&D Systems) or macrophage colony-stimulating factor (M-CSF, R&D Systems) and cultured in RPMI1640 medium with 10% FCS. Phycoerythrin (PE) conjugated monoclonal antibody against CD163 (556018, BD Biosciences) and CD206 (555954, BD Biosciences) were used to verify the phenotype and purity of differentiated MDMs as described [61].

### Immunoblot analysis for SAMHD1 degradation

HeLa cells stably expressing Flag-SAMHD1 were transfected with 4 μg of pCG-Vpx-EGFP expression constructs using polymer-based DNA trasnfection reagents (JetPEI™, Polyplus Transfection) following standard protocols of the manufacturer. At 48 h post-transfection cells were lysed in RIPA buffer containing protease inhibitor cocktail and then normalized for protein concentration. Whole-cell lysates containing 20 μg total proteins were separated on 4-12% Bis-Tris gradient acrylamide gels, transferred onto a polyvinylidene difluoride (PVDF) membrane and probed with a mixture of mouse monoclonal antibody to Flag (1:1000, Sigma) and Actin (1:2000, ab3280, Abcam) and rabbit polyclonal to GFP (1:1000, ab290, Abcam). Subsequently, blots were probed with anti-mouse or anti-rabbit IRDye Odyssey antibodies (926–32210, 926–32221). Proteins were revealed using a LI-COR Odyssey scanner and quantification of the signal intensities were analyzed by the Odyssey software. The ratios of SAMHD1 to ß-actin signal intensities were used to determine the efficiencies of Vpx-mediated SAMHD1 degradation.

### Virus production and MDMs transduction

Viral particles were produced from HEK 293T cells using the standard calcium phosphate transfection protocol [40]. For VSV-G HIV-1-eGFP production, 293T cells were transfected with 5 μg HIV-1 NL4-3 IRESeGFP and 0.8 μg VSV-G encoding plasmid; for Vpx incorporation, 1.2 μg pCG-Vpx-eGFP was co-transfected with 5 μg SIVmac239 ΔVpx-eGFP and 0.8 μg VSV-G encoding plasmid. Medium was replaced 16 h post-transfection and viruses were harvested 24 h later. The yield of infectious virus was determined by infection of TZM-bl indicator cells. Briefly, 8,000 cells were seeded in 96-well plate, after overnight incubation, cells were transduced with 100 μl VSV-G pseudotyped virus for 3 days and harvested for eGFP detection by flow cytometry using FACS CantoII. For MDMs transduction, 2.5 × 10^5^ cells were transduced with 800 μl VSV-G pseudotyped virus. After overnight incubation the medium was replaced with fresh medium and the MDMs were cultured for additional 4 days and infection rates were determined by flow cytometric detection of eGFP+ cells. The infectivity of each virus in MDMs was normalized to TZM-bl infectivity, the relative infection rate of SIVmac239 was set to 100%.

### Western blot

To monitor Vpx expression, HeLa or 293T cells were transfected with 4 μg of vector DNA co-expressing AU1-tagged Vpx and eGFP. Next day, cells were harvested and lysed in RIPA (1% NP-40, 0.5% Na-DOC, 0.1% SDS, 0.15 M NaCl, 50 mM Tris–HCl [pH 7.4], and 5 mM EDTA), and cell lysates were separated in 12% SDS-polyacrylamide (PAA) gels in a Tris-Tricine buffer system. After gel electrophoresis, proteins were transferred onto PVDF membranes and probed with AU1 (PRB-130P, Covance) and β-actin (8227–50, Abcam). For transfection control, another gel was run with similar conditions, after blotting the membrane was incubated with antibody specific for GFP (290–50, Abcam). Subsequently, blots were probed with anti-mouse or anti-rabbit IRDye Odyssey antibodies (926–32210, 926–32221) and proteins revealed using a LI-COR Odyssey scanner.

### Confocal microscopy

Immunofluorescence was performed as described earlier [62]. Briefly, HeLa cells stably expressing FLAG tagged SAMHD1 were cultivated on 8-well Ibidi slides (Ibidi GmbH). On the next day, cells were transfected with 150 ng of plasmid DNA expressing AU1 tagged Vpx. At 16–18 h post-transfection, cells were fixed with 4% paraformaldehyde for 30 min and stained by indirect immunofluorescence. After washing with phosphate-buffered saline (PBS), cells were treated with rabbit anti AU1 (1:500, Covance) and mouse MAb against FLAG tag (1:500, Abcam) in PBS containing 0.25% fish skin gelatin (Sigma), 0.2% bovine serum albumin (BSA), and 0.2% saponin for 2 h. Cells were washed again with PBS, incubated with Alexa Flour 568-conjugated goat anti-rabbit IgG and Alexa Flour 647-conjugated goat anti-mouse IgG (both 1:500 dilution; Invitrogen-Life Technologies) for another 2 h, washed again, and stained by Hoechst 33342. Images were acquired on an LSM 710 confocal microscope (Carl Zeiss, Germany) with Zeiss Zen 2010 software.

### Phylogenetic analysis

Vpx amino acid sequences were obtained from the Los Alamos HIV Sequence Database (http://hiv-web.lanl.gov). Phylogenetic trees were constructed using PhyML by the ML method (http://www.phylogeny.fr/version2_cgi/index.cgi).

### DCs activation and IFN-γ release assay

DCs were generated from PBMCs by 3 days stimulation with 50 ng/ml of rhGM-CSF and 25 ng/ml of IL-4 (R&D), and cultured in RPMI1640 medium with 10% FCS. Viral particles were produced from HEK 293T cells using the standard calcium phosphate transfection protocol. Briefly, 293T cells were transfected with 5 μg HIV-1 NL4-3 IRES-eGFP, SIVmac 239 IRES-eGFP, HIV-2 ROD IRES-eGFP and 0.8 μg VSV-G encoding plasmid. Medium was replaced 16 h post-transfection and viruses were harvest 24 h later. For DCs transduction, 2 × 10^5^ cells were transduced with 100 μl VSV-G pseudotyped virus, either alone or in combination. After 6 h incubation, the medium was replaced with fresh medium and the DCs were cultured for additional 72 hs. Infection rate was determined by detection of eGFP+ cells. DC activation was measured using the APC conjugated monoclonal antibody against CD86 (5397868, Invitrogen) by flow cytometry. Infectious supernatant was collected for measuring the interferon-γ release using the human IFN-γ ELISA kit, as described in the manufacturing handle book (Human IFN-γ ELISA Kit, RayBiotech).

### Ethics statement

Written informed consent was provided by all study participants or their legal guardians. Ethical approval was obtained from the Ethics Committee of the University Hospital Centre Rotterdam, Rotterdam, The Netherlands.

### Accession numbers

HIV-2 *vpx* sequences will be submitted to GenBank and accession numbers will be provided upon acceptance of the manuscript.

## Competing interests

The authors declare that they have no competing interests.

## Authors’ contributions

HY and SMU performed most of the experiments. AB, JK, CMS, MK, XL and DK also contributed experimental data and materials. MEE, ADO and RAG provided reagents, FK and HY conceived and coordinated the study and FK wrote the manuscript. All authors read and approved the final manuscript.

## Supplementary Material

Additional file 1: Figure S1 Redistribution and degradation of SAMHD1 by HIV-2 Vpx proteins. (A-K) Overview images from two different regions of HeLa cells stably expressing FLAG tagged SAMHD1, transfected with 150 ng of plasmid expressing the indicated AU1-tagged Vpx. Cells were stained for SAMHD1 (red), Vpx (green) and nucleus (blue) as described in the methods section. Scale bars represent 50 μm. Click here for file

Additional file 2: Figure S2 Redistribution and degradation of SAMHD1 by HIV-2 Vpx proteins. (A-H) HeLa cells stably expressing FLAG tagged SAMHD1 were transfected with 150 ng of plasmid expressing the indicated AU1-tagged Vpx. At 16–18 h post-transfection, cells were fixed and permeabilized and Vpx was detected using a rabbit anti AU1 and SAMHD1 was detected using a mouse anti-FLAG-tag, respectively. Scale bars represent 10 μm. Click here for file

Additional file 3: Figure S3 Effect of E15G and K68M substitutions on HIV-2 ROD Vpx localization and function. (A-C) HeLa cells were transfected with constructs expressing FLAG tagged SAMHD1 and the indicated AU1-tagged Vpx and analyzed as described in the legend to Figure 6. Scale bars represent 10 μm. Click here for file
