# Supplementary figures and images for: The efficiency of Vpx-mediated SAMHD1 antagonism does not correlate with the potency of viral control in HIV-2-infected individuals
Source: Retrovirology. 2013 Mar 5;10:27. doi: 10.1186/1742-4690-10-27 (PMC3599662; doi:10.1186/1742-4690-10-27)

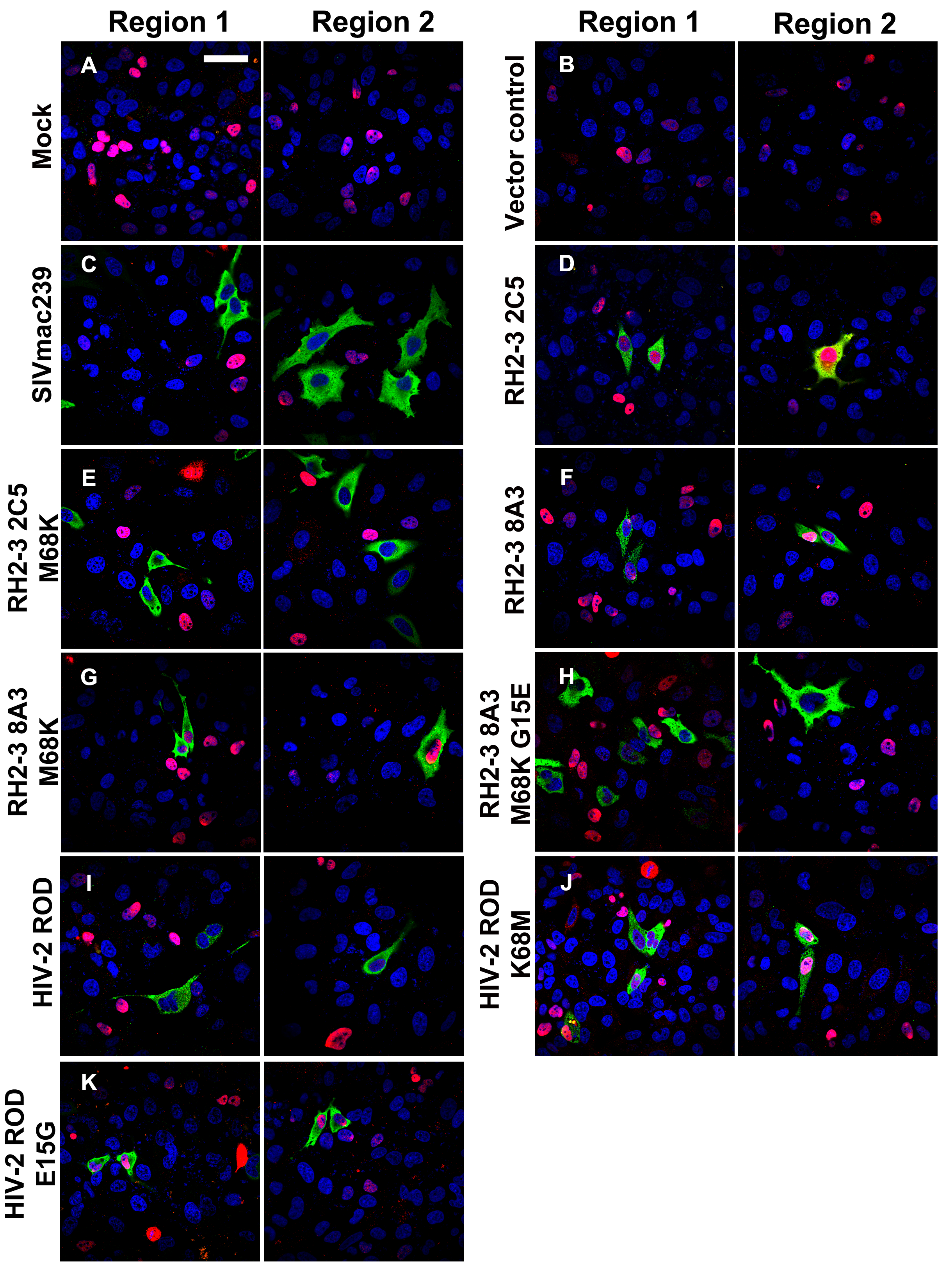

Supplement: Additional file 1: Figure S1 — Redistribution and degradation of SAMHD1 by HIV-2 Vpx proteins. (A-K) Overview images from two different regions of HeLa cells stably expressing FLAG tagged SAMHD1, transfected with 150 ng of plasmid expressing the indicated AU1-tagged Vpx. Cells were stained for SAMHD1 (red), Vpx (green) and nucleus (blue) as described in the methods section. Scale bars represent 50 μm. [file 1742-4690-10-27-S1.tiff]

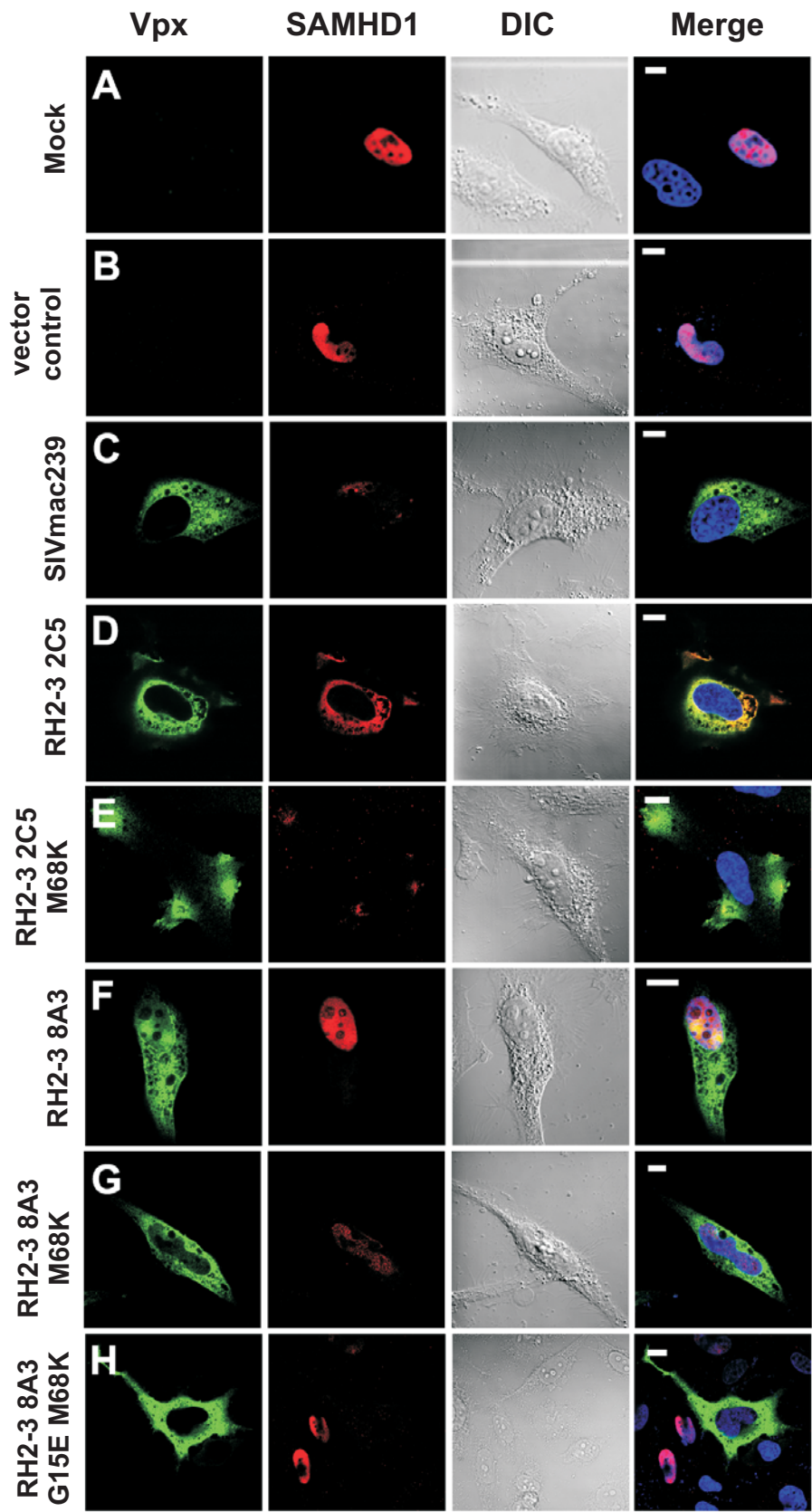

Supplement: Additional file 2: Figure S2 — Redistribution and degradation of SAMHD1 by HIV-2 Vpx proteins. (A-H) HeLa cells stably expressing FLAG tagged SAMHD1 were transfected with 150 ng of plasmid expressing the indicated AU1-tagged Vpx. At 16–18 h post-transfection, cells were fixed and permeabilized and Vpx was detected using a rabbit anti AU1 and SAMHD1 was detected using a mouse anti-FLAG-tag, respectively. Scale bars represent 10 μm. [file 1742-4690-10-27-S2.pdf]

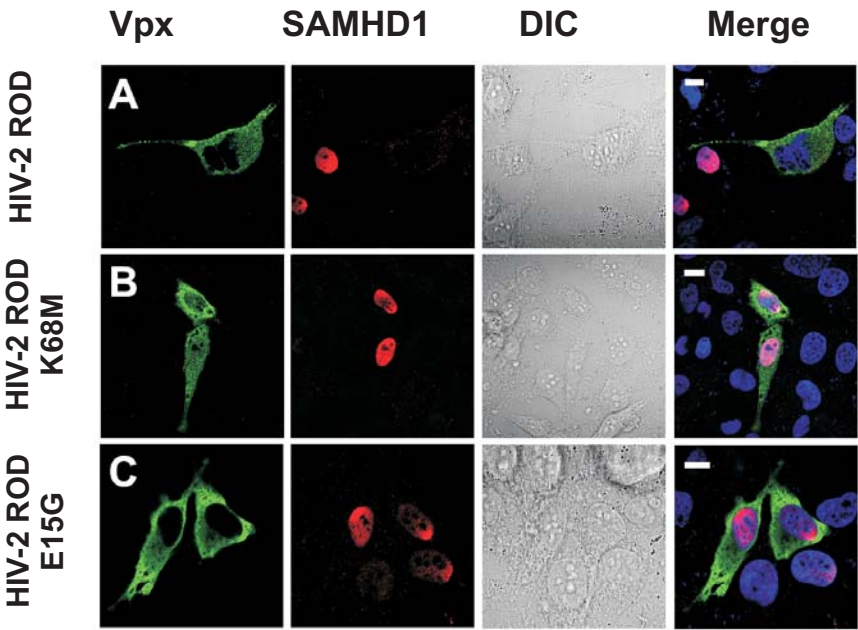

Supplement: Additional file 3: Figure S3 — Effect of E15G and K68M substitutions on HIV-2 ROD Vpx localization and function. (A-C) HeLa cells were transfected with constructs expressing FLAG tagged SAMHD1 and the indicated AU1-tagged Vpx and analyzed as described in the legend to Figure 6. Scale bars represent 10 μm. [file 1742-4690-10-27-S3.pdf]
